# Editorial: Cancer immunity and metabolic reprogramming: pioneering precision immunotherapies

**DOI:** 10.3389/fimmu.2026.1952020

**Published:** 2026-08-13

**Authors:** Haiyan He, Lifeng Feng, Jingyue Jia

**Affiliations:** 1 Department of Pharmacy, Sir Run Run Shaw Hospital, School of Medicine, Zhejiang University, Hangzhou, Zhejiang, China; 2 Department of Medical Oncology, Sir Run Run Shaw Hospital, School of Medicine, Zhejiang University, Hangzhou, Zhejiang, China; 3 Center for Global Health, Department of Internal Medicine, University of New Mexico Health Sciences Center, Albuquerque, NM, United States; 4 Autophagy, Inflammation and Metabolism Center of Biochemical Research Excellence, Albuquerque, NM, United States

**Keywords:** cancer immunity, cancer immunotherapy, immune evasion, metabolic reprogramming, tumor microenvironment

The clinical success of cancer immunotherapy has brought a new era in the treatment of malignant disease. By restoring and redirecting the patient’s own immune system to recognize and eliminate tumor cells, this approach has established a new treatment paradigm (1). Immune checkpoint inhibitors have produced durable responses in multiple malignancies, including melanoma, non-small cell lung cancer (NSCLC) and hepatocellular carcinoma (2), while adoptive cell therapies (3) and personalized cancer vaccines (4), have further expanded the scope of immune-based intervention. Nevertheless, the overall benefit of immunotherapy remains restricted. Only a subset of patients responds to treatment, and a considerable proportion of initial responders eventually develop resistance (5, 6). Accumulating evidence indicates that these outcomes are rarely determined by tumor-intrinsic features alone; instead, they are largely shaped by the immunosuppressive tumor microenvironment (TME) (7), in which impaired T-cell infiltration, cytotoxic T lymphocytes (CTLs) exhaustion, and the recruitment of immunosuppressive cells collectively lead to immune evasion and therapeutic failure (8, 9). Why antitumor immunity succeeds in some patients but fails in others, and how tumors construct and maintain the suppressive environment that underlies this variability, have therefore become two of the central challenges in cancer research.

Within the TME, CTLs face multiple barriers to infiltration and sustained effector function (8), whereas regulatory T cells (9), myeloid-derived suppressor cells (MDSCs) (10) and tumor-associated macrophages (TAMs) (11) actively maintain immune suppression, supported by soluble factors such as TGF-β, IL-10, and VEGF. Beyond this cellular composition, metabolic reprogramming is now recognized as a fundamental driver of immune dysfunction. Long regarded as a cell-intrinsic adaptation of tumor cells, metabolic rewiring is an established hallmark of cancer, enabling malignant cells to accelerate glycolysis, scavenge alternative carbon sources, and rewire lipid and amino acid metabolism under chronic stress (12, 13). Critically, these metabolic alterations are not confined to malignant cells. Through their metabolic activity, tumor and stromal cells collectively create a hostile biochemical environment in which infiltrating immune cells are chronically exposed to glucose deprivation, lactate accumulation, hypoxia, and disturbed lipid metabolism (14, 15). These constraints progressively disrupt antitumor immune competence and thus constitute a substantive mode of immune evasion, in which metabolic regulators form an interconnected network that coordinates the behavior of tumor and immune cells within the TME (13, 16).

These previous findings place the crosstalk between metabolic reprogramming and cancer immunity at the forefront of current research. Metabolic competition and metabolite-mediated signaling connect tumor progression with immune dysfunction in a self-reinforcing manner, and the metabolic interactions between tumor and immune cells may ultimately determine the efficacy of immunotherapy (17, 18). Recent technological advances, particularly single-cell transcriptomics and high-resolution metabolomics, now allow these interactions to be examined at unprecedented resolution, opening new avenues for identifying metabolic vulnerabilities for cancer immunotherapy (19). Deciphering this metabolic–immune crosstalk is therefore both a fundamental biological question and a pressing clinical need: it holds the key to overcoming resistance and advancing the next generation of precision immunotherapies. It is in this context that the present Research Topic, Cancer Immunity and Metabolic Reprogramming: Pioneering Precision Immunotherapies, was organized. This Research Topic gathers 11 contributions, including 7 original research articles, 3 review articles, and 1 brief research report, that collectively elucidate the metabolic–immune axis and propose strategies to improve cancer immunotherapy.

Innate immunity especially the cGAS/STING signaling pathway has been well defined as the crucial initiator of antitumor immunity, capable of enhancing antigen presenting to trigger adaptive immunity. Bai et al. investigated the natural compound baicalin in colorectal cancer and showed that it binds to hexokinase 2 (HK2) and promotes its proteasome dependent degradation. The resulting inhibition of glycolysis is accompanied by mitochondrial damage, which activates cGAS/STING signaling and drives tumor-associated macrophages toward an M1-like phenotype, thereby coupling tumor metabolic suppression with antitumor immune activation.

Metabolic intervention can likewise be beneficial in inflammation-driven carcinogenesis. Li et al. reported that D-allose suppresses tumor development in a colitis associated carcinogenesis model by restoring endoplasmic reticulum homeostasis and mitochondrial function in macrophages, while directly inhibiting colon cancer cell proliferation and migration. This dual action on immune re-activation and cancer metabolism supports D-allose as a candidate for preventing inflammation-associated tumorigenesis.

Beyond restraining tumor metabolism, glycolytic inhibition can also induce immunogenic cell death. An original study by Pan et al. demonstrated that 2-deoxy-D-glucose activates cAMP/PKA signaling, suppresses HK2, and triggers caspase-3/GSDME-dependent pyroptosis in breast cancer, with antitumor efficacy and acceptable toxicity *in vivo*. As pyroptosis is inherently immunogenic, the resulting cell rupture liberates damage associated molecular patterns and inflammatory mediators, which in turn activate antitumor immune responses.

The metabolic state of immune cells themselves is equally consequential. Speth et al. showed that lung tumor associated alveolar macrophages undergo a time dependent shift toward glycolysis that impairs SOCS3 secretion, a dysfunctional phenotype that can be reversed by limiting glycolytic flux. These findings position immune cell metabolism as a tractable target for relieving immunosuppression in non-small cell lung cancer.

In addition to serving as therapeutic targets, metabolic features can inform patient stratification. Zhao et al. identified a metabolically distinct malignant epithelial subpopulation in multiple primary lung cancers, marked by upregulated amino acid metabolism and MHC-II-mediated interactions with regulatory T cells and mast cells, and derived an eight-gene metabolic signature that stratifies survival and immunotherapy response potential in lung adenocarcinoma cohorts; the polyamine enzymes SMOX and SMS were functionally linked to tumor progression. In bladder cancer, Chen et al. connected lactate-driven protein lactylation to tumor behavior, identifying FASN and RUNX2 as hub genes whose knockdown reduces intracellular lactate and global protein lactylation, including prominent non-histone targets. Extending this theme to hematological malignancies, Wang et al. developed FAMscore, a machine learning-based signature of fatty acid metabolism genes in diffuse large B-cell lymphoma that independently predicts survival and is associated with an immunosuppressive infiltration profile, with CPT1A validated as a functional driver.

Host factors also warrant consideration. An original research report by Zidi et al. proposed a polygenic, immunogenetic framework for cervical cancer susceptibility based on a nine-SNP panel, providing hypothesis-generating evidence that germline variation may shape tumor immune behavior alongside metabolic features.

Three review articles place these findings in a broader context. Zhang et al. reviewed glucose metabolic reprogramming in pancreatic ductal adenocarcinoma, summarizing its contributions to driver gene regulation, mitochondrial dysfunction, microenvironmental remodeling, and chemoresistance, as well as emerging targeted intervention strategies. Dash et al. provided a comprehensive account of myeloid-derived suppressor cells, explaining how their metabolic reprogramming sustains immunosuppression and how metabolic inhibition, depletion, and differentiation-promoting strategies could be combined with checkpoint blockade and cell-based therapies. Looking toward newly recognized mechanisms, Xu et al. discussed glycosylated RNA as an interface between metabolism and immunity: sialylated GlycoRNAs engage Siglec receptors to promote immune escape, whereas partial deglycosylation exposes structures that activate Toll-like receptor-mediated innate immunity.

Taken together, the contributions in this Research Topic spanning original research, reviews, and a brief research report integrate metabolic and immunological layers of regulation, and establish metabolic reprogramming as a determinant of tumor progression, immune dysfunction, and therapeutic sensitivity. More importantly, they move the field beyond describing metabolic immune correlations toward defining causal, targetable mechanisms. We hope this Research Topic will serve as a valuable resource for researchers seeking to explore the crosstalk between metabolism and cancer immunity, develop new strategies that integrate metabolic intervention with metabolite-based, biomarker-guided stratification, and contribute to the realization of precision cancer immunotherapy.
